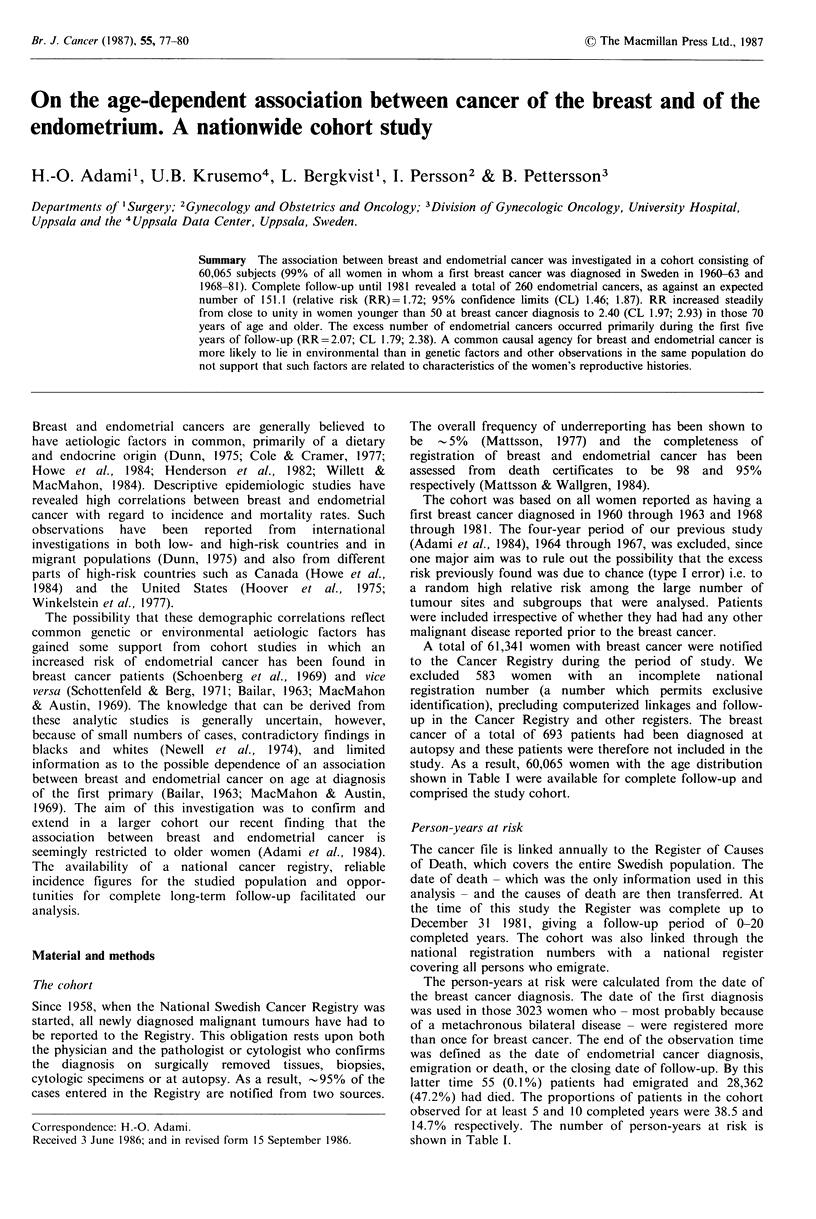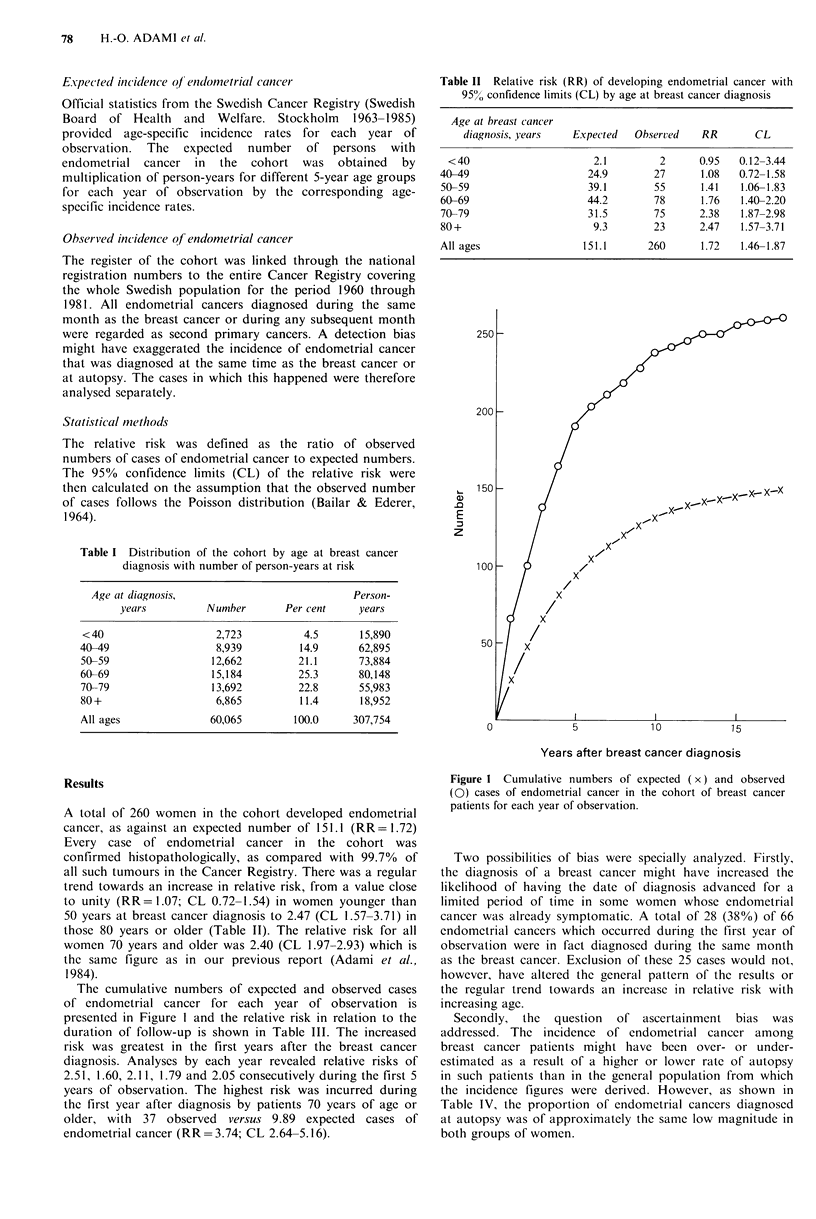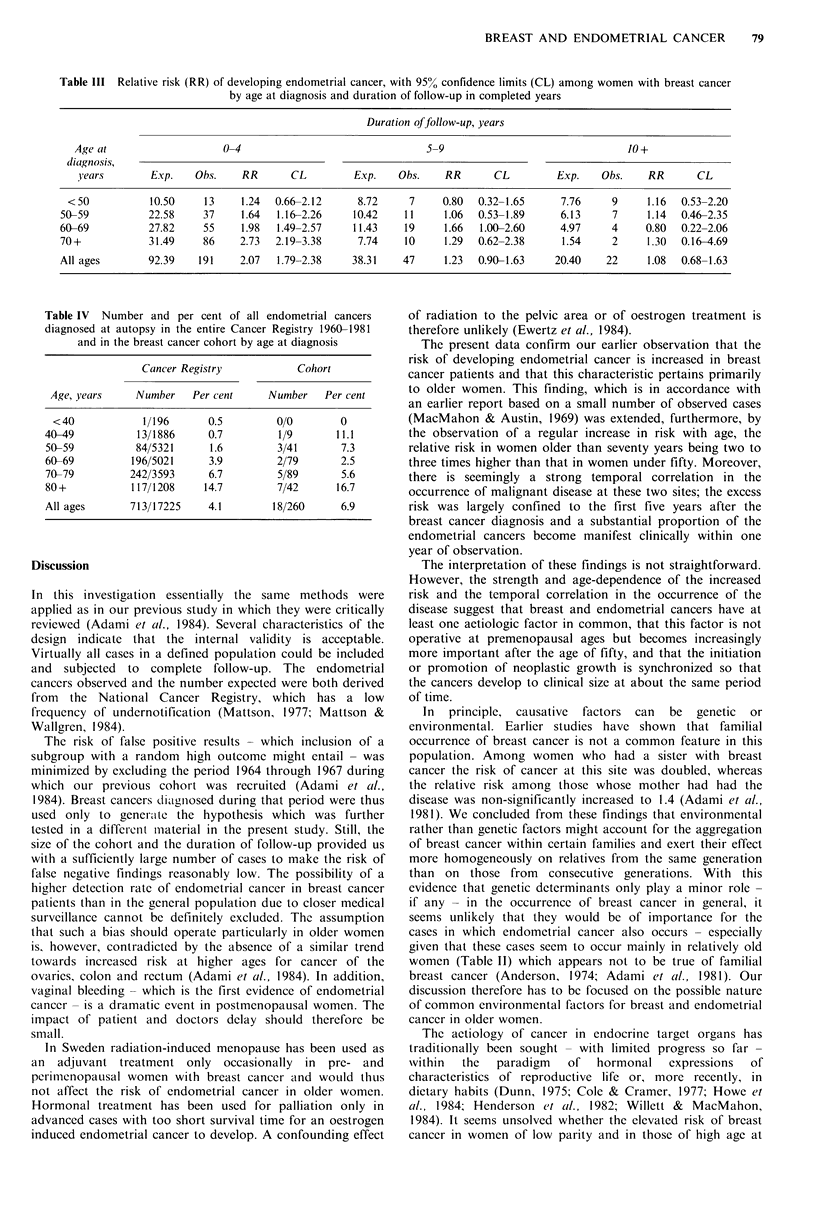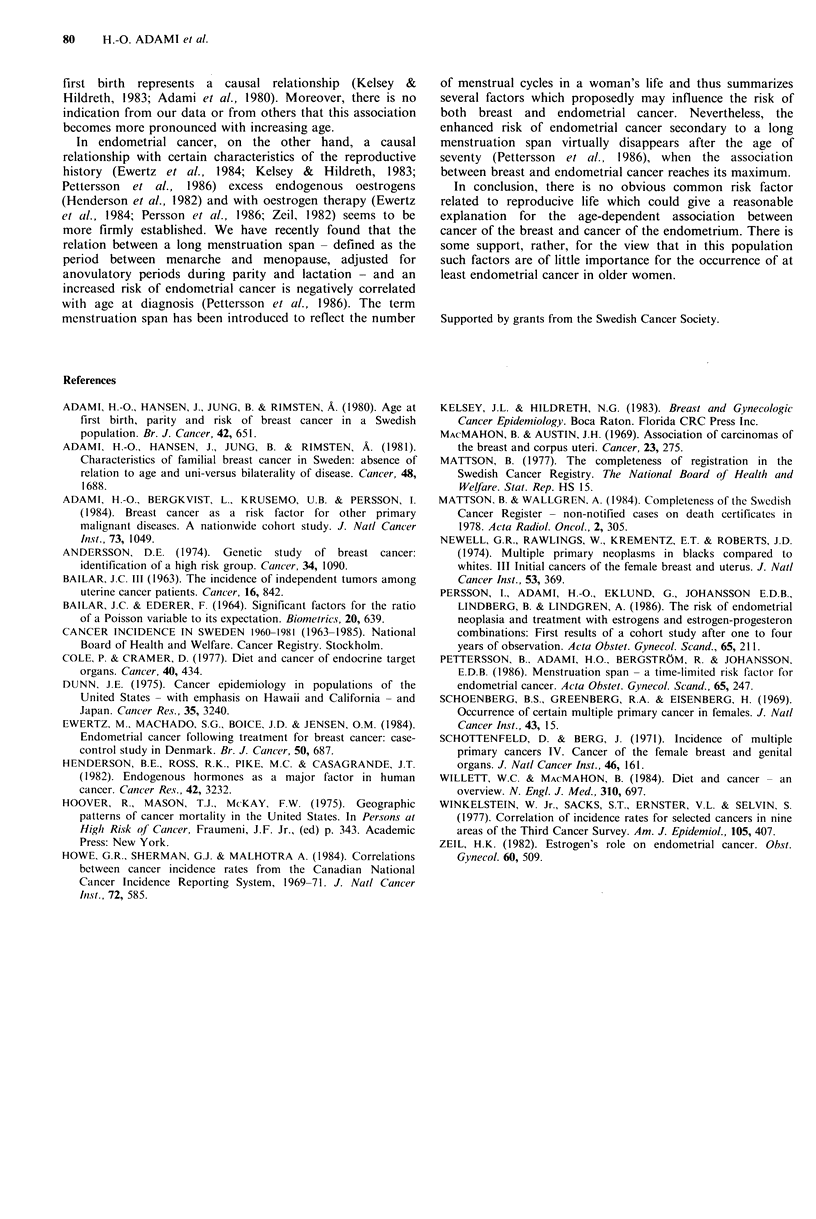# On the age-dependent association between cancer of the breast and of the endometrium. A nationwide cohort study.

**DOI:** 10.1038/bjc.1987.16

**Published:** 1987-01

**Authors:** H. O. Adami, U. B. Krusemo, L. Bergkvist, I. Persson, B. Pettersson

## Abstract

The association between breast and endometrial cancer was investigated in a cohort consisting of 60,065 subjects (99% of all women in whom a first breast cancer was diagnosed in Sweden in 1960-63 and 1968-81). Complete follow-up until 1981 revealed a total of 260 endometrial cancers, as against an expected number of 151.1 (relative risk (RR) = 1.72; 95% confidence limits (CL) 1.46; 1.87). RR increased steadily from close to unity in women younger than 50 at breast cancer diagnosis to 2.40 (CL 1.97; 2.93) in those 70 years of age and older. The excess number of endometrial cancers occurred primarily during the first five years of follow-up (RR = 2.07; CL 1.79; 2.38). A common causal agency for breast and endometrial cancer is more likely to lie in environmental than in genetic factors and other observations in the same population do not support that such factors are related to characteristics of the women's reproductive histories.


Br. J. Cancer (1987), 55, 77–80 © The Macmillan Press Ltd., 1987

### On the age-dependent association between cancer of the breast and of the endometrium. A nationwide cohort study

H.-O. Adami[1], U.B. Krusemo[4], L. Bergkvist[1], I. Persson[2] & B. Pettersson[3]

*Departments of [1]Surgery; [2]Gynecology and Obstetrics and Oncology; [3]Division of Gynecologic Oncology, University Hospital, Uppsala and the [4]Uppsala Data Center, Uppsala, Sweden.*

**Summary** The association between breast and endometrial cancer was investigated in a cohort consisting of 60,065 subjects (99% of all women in whom a first breast cancer was diagnosed in Sweden in 1960–63 and 1968–81). Complete follow-up until 1981 revealed a total of 260 endometrial cancers, as against an expected number of 151.1 (relative risk (RR) = 1.72; 95% confidence limits (CL) 1.46; 1.87). RR increased steadily from close to unity in women younger than 50 at breast cancer diagnosis to 2.40 (CL 1.97; 2.93) in those 70 years of age and older. The excess number of endometrial cancers occurred primarily during the first five years of follow-up (RR = 2.07; CL 1.79; 2.38). A common causal agency for breast and endometrial cancer is more likely to lie in environmental than in genetic factors and other observations in the same population do not support that such factors are related to characteristics of the women's reproductive histories.

Breast and endometrial cancers are generally believed to have aetiologic factors in common, primarily of a dietary and endocrine origin (Dunn, 1975; Cole & Cramer, 1977; Howe *et al.*, 1984; Henderson *et al.*, 1982; Willett & MacMahon, 1984). Descriptive epidemiologic studies have revealed high correlations between breast and endometrial cancer with regard to incidence and mortality rates. Such observations have been reported from international investigations in both low- and high-risk countries and in migrant populations (Dunn, 1975) and also from different parts of high-risk countries such as Canada (Howe *et al.*, 1984) and the United States (Hoover *et al.*, 1975; Winkelstein *et al.*, 1977).

The possibility that these demographic correlations reflect common genetic or environmental aetiologic factors has gained some support from cohort studies in which an increased risk of endometrial cancer has been found in breast cancer patients (Schoenberg *et al.*, 1969) and *vice versa* (Schottenfeld & Berg, 1971; Bailar, 1963; MacMahon & Austin, 1969). The knowledge that can be derived from these analytic studies is generally uncertain, however, because of small numbers of cases, contradictory findings in blacks and whites (Newell *et al.*, 1974), and limited information as to the possible dependence of an association between breast and endometrial cancer on age at diagnosis of the first primary (Bailar, 1963; MacMahon & Austin, 1969). The aim of this investigation was to confirm and extend in a larger cohort our recent finding that the association between breast and endometrial cancer is seemingly restricted to older women (Adami *et al.*, 1984). The availability of a national cancer registry, reliable incidence figures for the studied population and opportunities for complete long-term follow-up facilitated our analysis.

### Material and methods

### *The cohort*

Since 1958, when the National Swedish Cancer Registry was started, all newly diagnosed malignant tumours have had to be reported to the Registry. This obligation rests upon both the physician and the pathologist or cytologist who confirms the diagnosis on surgically removed tissues, biopsies, cytologic specimens or at autopsy. As a result, ~95% of the cases entered in the Registry are notified from two sources. The overall frequency of underreporting has been shown to be ~5% (Mattsson, 1977) and the completeness of registration of breast and endometrial cancer has been assessed from death certificates to be 98 and 95% respectively (Mattsson & Wallgren, 1984).

The cohort was based on all women reported as having a first breast cancer diagnosed in 1960 through 1963 and 1968 through 1981. The four-year period of our previous study (Adami *et al.*, 1984), 1964 through 1967, was excluded, since one major aim was to rule out the possibility that the excess risk previously found was due to chance (type I error) i.e. to a random high relative risk among the large number of tumour sites and subgroups that were analysed. Patients were included irrespective of whether they had had any other malignant disease reported prior to the breast cancer.

A total of 61,341 women with breast cancer were notified to the Cancer Registry during the period of study. We excluded 583 women with an incomplete national registration number (a number which permits exclusive identification), precluding computerized linkages and follow-up in the Cancer Registry and other registers. The breast cancer of a total of 693 patients had been diagnosed at autopsy and these patients were therefore not included in the study. As a result, 60,065 women with the age distribution shown in Table I were available for complete follow-up and comprised the study cohort.

### *Person-years at risk*

The cancer file is linked annually to the Register of Causes of Death, which covers the entire Swedish population. The date of death – which was the only information used in this analysis – and the causes of death are then transferred. At the time of this study the Register was complete up to December 31 1981, giving a follow-up period of 0–20 completed years. The cohort was also linked through the national registration numbers with a national register covering all persons who emigrate.

The person-years at risk were calculated from the date of the breast cancer diagnosis. The date of the first diagnosis was used in those 3023 women who – most probably because of a metachronous bilateral disease – were registered more than once for breast cancer. The end of the observation time was defined as the date of endometrial cancer diagnosis, emigration or death, or the closing date of follow-up. By this latter time 55 (0.1%) patients had emigrated and 28,362 (47.2%) had died. The proportions of patients in the cohort observed for at least 5 and 10 completed years were 38.5 and 14.7% respectively. The number of person-years at risk is shown in Table I.

Correspondence: H.-O. Adami.
Received 3 June 1986; and in revised form 15 September 1986.

*Expected incidence of endometrial cancer*

Official statistics from the Swedish Cancer Registry (Swedish Board of Health and Welfare. Stockholm 1963–1985) provided age-specific incidence rates for each year of observation. The expected number of persons with endometrial cancer in the cohort was obtained by multiplication of person-years for different 5-year age groups for each year of observation by the corresponding age-specific incidence rates.

*Observed incidence of endometrial cancer*

The register of the cohort was linked through the national registration numbers to the entire Cancer Registry covering the whole Swedish population for the period 1960 through 1981. All endometrial cancers diagnosed during the same month as the breast cancer or during any subsequent month were regarded as second primary cancers. A detection bias might have exaggerated the incidence of endometrial cancer that was diagnosed at the same time as the breast cancer or at autopsy. The cases in which this happened were therefore analysed separately.

*Statistical methods*

The relative risk was defined as the ratio of observed numbers of cases of endometrial cancer to expected numbers. The 95% confidence limits (CL) of the relative risk were then calculated on the assumption that the observed number of cases follows the Poisson distribution (Bailar & Ederer, 1964).

**Table I** Distribution of the cohort by age at breast cancer diagnosis with number of person-years at risk

| *Age at diagnosis, years* | *Number* | *Per cent* | *Person-years* |
|---|---|---|---|
| <40 | 2,723 | 4.5 | 15,890 |
| 40–49 | 8,939 | 14.9 | 62,895 |
| 50–59 | 12,662 | 21.1 | 73,884 |
| 60–69 | 15,184 | 25.3 | 80,148 |
| 70–79 | 13,692 | 22.8 | 55,983 |
| 80+ | 6,865 | 11.4 | 18,952 |
| All ages | 60,065 | 100.0 | 307,754 |

### Results

A total of 260 women in the cohort developed endometrial cancer, as against an expected number of 151.1 (RR = 1.72) Every case of endometrial cancer in the cohort was confirmed histopathologically, as compared with 99.7% of all such tumours in the Cancer Registry. There was a regular trend towards an increase in relative risk, from a value close to unity (RR = 1.07; CL 0.72–1.54) in women younger than 50 years at breast cancer diagnosis to 2.47 (CL 1.57–3.71) in those 80 years or older (Table II). The relative risk for all women 70 years and older was 2.40 (CL 1.97–2.93) which is the same figure as in our previous report (Adami *et al.*, 1984).

The cumulative numbers of expected and observed cases of endometrial cancer for each year of observation is presented in Figure 1 and the relative risk in relation to the duration of follow-up is shown in Table III. The increased risk was greatest in the first years after the breast cancer diagnosis. Analyses by each year revealed relative risks of 2.51, 1.60, 2.11, 1.79 and 2.05 consecutively during the first 5 years of observation. The highest risk was incurred during the first year after diagnosis by patients 70 years of age or older, with 37 observed *versus* 9.89 expected cases of endometrial cancer (RR = 3.74; CL 2.64–5.16).

**Table II** Relative risk (RR) of developing endometrial cancer with 95% confidence limits (CL) by age at breast cancer diagnosis

| *Age at breast cancer diagnosis, years* | *Expected* | *Observed* | *RR* | *CL* |
|---|---|---|---|---|
| <40 | 2.1 | 2 | 0.95 | 0.12–3.44 |
| 40–49 | 24.9 | 27 | 1.08 | 0.72–1.58 |
| 50–59 | 39.1 | 55 | 1.41 | 1.06–1.83 |
| 60–69 | 44.2 | 78 | 1.76 | 1.40–2.20 |
| 70–79 | 31.5 | 75 | 2.38 | 1.87–2.98 |
| 80+ | 9.3 | 23 | 2.47 | 1.57–3.71 |
| All ages | 151.1 | 260 | 1.72 | 1.46–1.87 |

**Figure 1** Cumulative numbers of expected (×) and observed (○) cases of endometrial cancer in the cohort of breast cancer patients for each year of observation.

Two possibilities of bias were specially analyzed. Firstly, the diagnosis of a breast cancer might have increased the likelihood of having the date of diagnosis advanced for a limited period of time in some women whose endometrial cancer was already symptomatic. A total of 28 (38%) of 66 endometrial cancers which occurred during the first year of observation were in fact diagnosed during the same month as the breast cancer. Exclusion of these 25 cases would not, however, have altered the general pattern of the results or the regular trend towards an increase in relative risk with increasing age.

Secondly, the question of ascertainment bias was addressed. The incidence of endometrial cancer among breast cancer patients might have been over- or underestimated as a result of a higher or lower rate of autopsy in such patients than in the general population from which the incidence figures were derived. However, as shown in Table IV, the proportion of endometrial cancers diagnosed at autopsy was of approximately the same low magnitude in both groups of women.

**Table III** Relative risk (RR) of developing endometrial cancer, with 95% confidence limits (CL) among women with breast cancer by age at diagnosis and duration of follow-up in completed years

| | *Duration of follow-up, years* | | | | | | | | | | | |
|---|---|---|---|---|---|---|---|---|---|---|---|---|
| *Age at diagnosis, years* | *0–4* | | | | *5–9* | | | | *10+* | | | |
| | *Exp.* | *Obs.* | *RR* | *CL* | *Exp.* | *Obs.* | *RR* | *CL* | *Exp.* | *Obs.* | *RR* | *CL* |
| <50 | 10.50 | 13 | 1.24 | 0.66–2.12 | 8.72 | 7 | 0.80 | 0.32–1.65 | 7.76 | 9 | 1.16 | 0.53–2.20 |
| 50–59 | 22.58 | 37 | 1.64 | 1.16–2.26 | 10.42 | 11 | 1.06 | 0.53–1.89 | 6.13 | 7 | 1.14 | 0.46–2.35 |
| 60–69 | 27.82 | 55 | 1.98 | 1.49–2.57 | 11.43 | 19 | 1.66 | 1.00–2.60 | 4.97 | 4 | 0.80 | 0.22–2.06 |
| 70+ | 31.49 | 86 | 2.73 | 2.19–3.38 | 7.74 | 10 | 1.29 | 0.62–2.38 | 1.54 | 2 | 1.30 | 0.16–4.69 |
| All ages | 92.39 | 191 | 2.07 | 1.79–2.38 | 38.31 | 47 | 1.23 | 0.90–1.63 | 20.40 | 22 | 1.08 | 0.68–1.63 |

**Table IV** Number and per cent of all endometrial cancers diagnosed at autopsy in the entire Cancer Registry 1960–1981 and in the breast cancer cohort by age at diagnosis

| | *Cancer Registry* | | *Cohort* | |
|---|---|---|---|---|
| *Age, years* | *Number* | *Per cent* | *Number* | *Per cent* |
| <40 | 1/196 | 0.5 | 0/0 | 0 |
| 40–49 | 13/1886 | 0.7 | 1/9 | 11.1 |
| 50–59 | 84/5321 | 1.6 | 3/41 | 7.3 |
| 60–69 | 196/5021 | 3.9 | 2/79 | 2.5 |
| 70–79 | 242/3593 | 6.7 | 5/89 | 5.6 |
| 80+ | 117/1208 | 14.7 | 7/42 | 16.7 |
| All ages | 713/17225 | 4.1 | 18/260 | 6.9 |

### Discussion

In this investigation essentially the same methods were applied as in our previous study in which they were critically reviewed (Adami *et al.*, 1984). Several characteristics of the design indicate that the internal validity is acceptable. Virtually all cases in a defined population could be included and subjected to complete follow-up. The endometrial cancers observed and the number expected were both derived from the National Cancer Registry, which has a low frequency of undernotification (Mattson, 1977; Mattson & Wallgren, 1984).

The risk of false positive results – which inclusion of a subgroup with a random high outcome might entail – was minimized by excluding the period 1964 through 1967 during which our previous cohort was recruited (Adami *et al.*, 1984). Breast cancers diagnosed during that period were thus used only to generate the hypothesis which was further tested in a different material in the present study. Still, the size of the cohort and the duration of follow-up provided us with a sufficiently large number of cases to make the risk of false negative findings reasonably low. The possibility of a higher detection rate of endometrial cancer in breast cancer patients than in the general population due to closer medical surveillance cannot be definitely excluded. The assumption that such a bias should operate particularly in older women is, however, contradicted by the absence of a similar trend towards increased risk at higher ages for cancer of the ovaries, colon and rectum (Adami *et al.*, 1984). In addition, vaginal bleeding – which is the first evidence of endometrial cancer – is a dramatic event in postmenopausal women. The impact of patient and doctors delay should therefore be small.

In Sweden radiation-induced menopause has been used as an adjuvant treatment only occasionally in pre- and perimenopausal women with breast cancer and would thus not affect the risk of endometrial cancer in older women. Hormonal treatment has been used for palliation only in advanced cases with too short survival time for an oestrogen induced endometrial cancer to develop. A confounding effect of radiation to the pelvic area or of oestrogen treatment is therefore unlikely (Ewertz *et al.*, 1984).

The present data confirm our earlier observation that the risk of developing endometrial cancer is increased in breast cancer patients and that this characteristic pertains primarily to older women. This finding, which is in accordance with an earlier report based on a small number of observed cases (MacMahon & Austin, 1969) was extended, furthermore, by the observation of a regular increase in risk with age, the relative risk in women older than seventy years being two to three times higher than that in women under fifty. Moreover, there is seemingly a strong temporal correlation in the occurrence of malignant disease at these two sites; the excess risk was largely confined to the first five years after the breast cancer diagnosis and a substantial proportion of the endometrial cancers become manifest clinically within one year of observation.

The interpretation of these findings is not straightforward. However, the strength and age-dependence of the increased risk and the temporal correlation in the occurrence of the disease suggest that breast and endometrial cancers have at least one aetiologic factor in common, that this factor is not operative at premenopausal ages but becomes increasingly more important after the age of fifty, and that the initiation or promotion of neoplastic growth is synchronized so that the cancers develop to clinical size at about the same period of time.

In principle, causative factors can be genetic or environmental. Earlier studies have shown that familial occurrence of breast cancer is not a common feature in this population. Among women who had a sister with breast cancer the risk of cancer at this site was doubled, whereas the relative risk among those whose mother had had the disease was non-significantly increased to 1.4 (Adami *et al.*, 1981). We concluded from these findings that environmental rather than genetic factors might account for the aggregation of breast cancer within certain families and exert their effect more homogeneously on relatives from the same generation than on those from consecutive generations. With this evidence that genetic determinants only play a minor role – if any – in the occurrence of breast cancer in general, it seems unlikely that they would be of importance for the cases in which endometrial cancer also occurs – especially given that these cases seem to occur mainly in relatively old women (Table II) which appears not to be true of familial breast cancer (Anderson, 1974; Adami *et al.*, 1981). Our discussion therefore has to be focused on the possible nature of common environmental factors for breast and endometrial cancer in older women.

The aetiology of cancer in endocrine target organs has traditionally been sought – with limited progress so far – within the paradigm of hormonal expressions of characteristics of reproductive life or, more recently, in dietary habits (Dunn, 1975; Cole & Cramer, 1977; Howe *et al.*, 1984; Henderson *et al.*, 1982; Willett & MacMahon, 1984). It seems unsolved whether the elevated risk of breast cancer in women of low parity and in those of high age at first birth represents a causal relationship (Kelsey & Hildreth, 1983; Adami *et al.*, 1980). Moreover, there is no indication from our data or from others that this association becomes more pronounced with increasing age.

In endometrial cancer, on the other hand, a causal relationship with certain characteristics of the reproductive history (Ewertz *et al.*, 1984; Kelsey & Hildreth, 1983; Pettersson *et al.*, 1986) excess endogenous oestrogens (Henderson *et al.*, 1982) and with oestrogen therapy (Ewertz *et al.*, 1984; Persson *et al.*, 1986; Zeil, 1982) seems to be more firmly established. We have recently found that the relation between a long menstruation span – defined as the period between menarche and menopause, adjusted for anovulatory periods during parity and lactation – and an increased risk of endometrial cancer is negatively correlated with age at diagnosis (Pettersson *et al.*, 1986). The term menstruation span has been introduced to reflect the number of menstrual cycles in a woman's life and thus summarizes several factors which proposedly may influence the risk of both breast and endometrial cancer. Nevertheless, the enhanced risk of endometrial cancer secondary to a long menstruation span virtually disappears after the age of seventy (Pettersson *et al.*, 1986), when the association between breast and endometrial cancer reaches its maximum.

In conclusion, there is no obvious common risk factor related to reproducive life which could give a reasonable explanation for the age-dependent association between cancer of the breast and cancer of the endometrium. There is some support, rather, for the view that in this population such factors are of little importance for the occurrence of at least endometrial cancer in older women.

Supported by grants from the Swedish Cancer Society.

**References**

ADAMI, H.-O., HANSEN, J., JUNG, B. & RIMSTEN, Å. (1980). Age at first birth, parity and risk of breast cancer in a Swedish population. *Br. J. Cancer*, **42**, 651.

ADAMI, H.-O., HANSEN, J., JUNG, B. & RIMSTEN, Å. (1981). Characteristics of familial breast cancer in Sweden: absence of relation to age and uni-versus bilaterality of disease. *Cancer*, **48**, 1688.

ADAMI, H.-O., BERGKVIST, L., KRUSEMO, U.B. & PERSSON, I. (1984). Breast cancer as a risk factor for other primary malignant diseases. A nationwide cohort study. *J. Natl Cancer Inst.*, **73**, 1049.

ANDERSSON, D.E. (1974). Genetic study of breast cancer: identification of a high risk group. *Cancer*, **34**, 1090.

BAILAR, J.C. III (1963). The incidence of independent tumors among uterine cancer patients. *Cancer*, **16**, 842.

BAILAR, J.C. & EDERER, F. (1964). Significant factors for the ratio of a Poisson variable to its expectation. *Biometrics*, **20**, 639.

CANCER INCIDENCE IN SWEDEN 1960–1981 (1963–1985). National Board of Health and Welfare. Cancer Registry. Stockholm.

COLE, P. & CRAMER, D. (1977). Diet and cancer of endocrine target organs. *Cancer*, **40**, 434.

DUNN, J.E. (1975). Cancer epidemiology in populations of the United States – with emphasis on Hawaii and California – and Japan. *Cancer Res.*, **35**, 3240.

EWERTZ, M., MACHADO, S.G., BOICE, J.D. & JENSEN, O.M. (1984). Endometrial cancer following treatment for breast cancer: case-control study in Denmark. *Br. J. Cancer*, **50**, 687.

HENDERSON, B.E., ROSS, R.K., PIKE, M.C. & CASAGRANDE, J.T. (1982). Endogenous hormones as a major factor in human cancer. *Cancer Res.*, **42**, 3232.

HOOVER, R., MASON, T.J., McKAY, F.W. (1975). Geographic patterns of cancer mortality in the United States. In *Persons at High Risk of Cancer*, Fraumeni, J.F. Jr., (ed) p. 343. Academic Press: New York.

HOWE, G.R., SHERMAN, G.J. & MALHOTRA A. (1984). Correlations between cancer incidence rates from the Canadian National Cancer Incidence Reporting System, 1969–71. *J. Natl Cancer Inst.*, **72**, 585.

KELSEY, J.L. & HILDRETH, N.G. (1983). *Breast and Gynecologic Cancer Epidemiology*. Boca Raton. Florida CRC Press Inc.

MacMAHON, B. & AUSTIN, J.H. (1969). Association of carcinomas of the breast and corpus uteri. *Cancer*, **23**, 275.

MATTSON, B. (1977). The completeness of registration in the Swedish Cancer Registry. *The National Board of Health and Welfare. Stat. Rep.* HS 15.

MATTSON, B. & WALLGREN, A. (1984). Completeness of the Swedish Cancer Register – non-notified cases on death certificates in 1978. *Acta Radiol. Oncol.*, **2**, 305.

NEWELL, G.R., RAWLINGS, W., KREMENTZ, E.T. & ROBERTS, J.D. (1974). Multiple primary neoplasms in blacks compared to whites. III Initial cancers of the female breast and uterus. *J. Natl Cancer Inst.*, **53**, 369.

PERSSON, I., ADAMI, H.-O., EKLUND, G., JOHANSSON E.D.B., LINDBERG, B. & LINDGREN, A. (1986). The risk of endometrial neoplasia and treatment with estrogens and estrogen-progesteron combinations: First results of a cohort study after one to four years of observation. *Acta Obstet. Gynecol. Scand.*, **65**, 211.

PETTERSSON, B., ADAMI, H.O., BERGSTRÖM, R. & JOHANSSON, E.D.B. (1986). Menstruation span – a time-limited risk factor for endometrial cancer. *Acta Obstet. Gynecol. Scand.*, **65**, 247.

SCHOENBERG, B.S., GREENBERG, R.A. & EISENBERG, H. (1969). Occurrence of certain multiple primary cancer in females. *J. Natl Cancer Inst.*, **43**, 15.

SCHOTTENFELD, D. & BERG, J. (1971). Incidence of multiple primary cancers IV. Cancer of the female breast and genital organs. *J. Natl Cancer Inst.*, **46**, 161.

WILLETT, W.C. & MacMAHON, B. (1984). Diet and cancer – an overview. *N. Engl. J. Med.*, **310**, 697.

WINKELSTEIN, W. Jr., SACKS, S.T., ERNSTER, V.L. & SELVIN, S. (1977). Correlation of incidence rates for selected cancers in nine areas of the Third Cancer Survey. *Am. J. Epidemiol.*, **105**, 407.

ZEIL, H.K. (1982). Estrogen's role on endometrial cancer. *Obst. Gynecol.* **60**, 509.